# Biological correlates of treatment resistant depression: a review of peripheral biomarkers

**DOI:** 10.3389/fpsyt.2023.1291176

**Published:** 2023-10-24

**Authors:** Emiliana Mancuso, Gaia Sampogna, Alessia Boiano, Bianca Della Rocca, Matteo Di Vincenzo, Maria Vita Lapadula, Flavia Martinelli, Federico Lucci, Mario Luciano

**Affiliations:** Department of Psychiatry, University of Campania “L. Vanvitelli”, Caserta, Italy

**Keywords:** major depression, treatment resistant depression, TRD, biomarker, cytokines, inflammation

## Abstract

**Introduction:**

Many patients fail to respond to multiple antidepressant interventions, being defined as “treatment-resistant depression” (TRD) patients. TRD is usually associated with increased severity and chronicity of symptoms, increased risk of comorbidity, and higher suicide rates, which make the clinical management challenging. Efforts to distinguish between TRD patients and those who will respond to treatment have been unfruitful so far. Several studies have tried to identify the biological, psychopathological, and psychosocial correlates of depression, with particular attention to the inflammatory system. In this paper we aim to review available studies assessing the full range of biomarkers in TRD patients in order to reshape TRD definition and improve its diagnosis, treatment, and prognosis.

**Methods:**

We searched the most relevant medical databases and included studies reporting original data on possible biomarkers of TRD. The keywords “treatment resistant depression” or “TRD” matched with “biomarker,” “inflammation,” “hormone,” “cytokine” or “biological marker” were entered in PubMed, ISI Web of Knowledge and SCOPUS databases. Articles were included if they included a comparison with healthy controls (HC).

**Results:**

Of the 1878 papers identified, 35 were included in the present study. Higher plasma levels of IL-6 and TNF-α were detected in TRD patients compared to HC. While only a few studies on cortisol have been found, four papers showed elevated levels of C-reactive protein among these patients and four articles focused on immunological cells. Altered kynurenine metabolism in TRD patients was reported in two studies, while contrasting results were found with regard to BDNF.

**Conclusion:**

Only a few biological alterations correlate with TRD. TNF-α seems to be the most relevant biomarker to discriminate TRD patients from both HC and treatment-responsive MDD patients. Moreover, several discrepancies among studies have been found, due to methodological differences and the lack of a standardized diagnostic definition of TRD.

## Introduction

Major Depressive Disorder (MDD) is a heterogeneous severe mental disorder, deriving from the interplay between genetic, environmental and psychological factors (1). More than 280 million people suffer from MDD, which is the primary cause of disability worldwide (2) and of significant impairment in daily functioning and quality of life (3, 4). At least 80% of patients with MDD experience work difficulties, problematic social interactions, and impaired daily life activities, making difficult the achievement of a full functional recovery (5, 6). Several effective pharmacological and psychosocial interventions are available for MDD, but many patients fail to respond to multiple antidepressant interventions, being defined as “treatment-resistant depression” (TRD) patients (7).

The first conceptualization of TRD dates back to 1970s as an attempt to overcome the limitation of the construct of “refractory depression” (8). Subsequently, Ban (9) argued that failure to respond to pharmacological treatment in patients with depression might reflect a different neurobiological substrate of depressive symptoms, compared to those patients who responded adequately to antidepressants. Accordingly, resistance to antidepressants would define for a distinct clinical subtype of depression. The first clinical definition of TRD was provided only in the late 90s by Thase and Rush (10), who described a sample of depressed patients who had not responded to at least two adequate trials of antidepressant medications, revitalizing the concept of TRD. Since then, the concept of TRD has been constantly refined (11, 12).

Currently, different definitions of TRD are available. The European Medicines Agency (EMA) defined resistance as a “failure to produce significant clinical results with a treatment of at least two different antidepressants (of the same or different classes) administered at the right doses and for an adequate amount of time, with verified patients’ compliance to treatment,” and is widely adopted as a standard definition of TRD in research settings (13). According to the Maudsley Staging Method, TRD is defined by five domains: time-course, severity, number of drugs, augmentative strategies, and use of ECT, with a maximum score of 15 (14). However, despite efforts, the definition of treatment resistant depression still presents several critical issues. In fact, some authors pointed out that the resistance construct can lead to a sense of nihilism in both patients and mental health professionals (15), and the construct of Difficult-To-Treat Depression (DTTD) would be preferable: while TRD focuses on a trial-and-error approach to find the right treatment, DTTD recognizes the importance of tailoring treatment to the needs of individual patients and considers a more comprehensive evaluation of patient’s medical history, lifestyle, and other subjective variables (16, 17). However, more complex and accurate definitions are poorly represented in clinical trials (18).

The difficulties in increasing knowledge about epidemiology, clinical management, and treatment of TRD are partially due to the lack of a univocal definition of this syndrome, which is highly needed. In fact, resistance to antidepressants is associated with greater symptom severity and chronicity, increased risk of comorbid physical (19, 20) and mental disorders, and higher suicide rates (21). Thus, TRD might represent a distinct clinical subtype of depression, yet one of the more severe, with unique treatment challenges and implications (22, 23), or a more severe form of MDD at the extreme of the affective continuum.

In order to gain deeper insights into the presence of a distinct clinical phenotype of TRD with discernible biological foundations, in this paper we have investigated biomarkers, specifically those previously documented in the literature for their associations with TRD. Biomarker can be defined as “A defined characteristic that is measured as an indicator of normal biological processes, pathogenic processes or responses to an exposure or intervention” (24). Biomarkers, as measurable molecular or cellular indicators, hold the potential to unravel the intricate interplay between genetic, physiological, and environmental factors that contribute to the manifestation of unique clinical profiles. These biomarkers serve as invaluable tools, facilitating the characterization, diagnosis, and understanding of the underlying biological mechanisms associated with a specific clinical phenotype. In the field of psychiatry, the practical application of biomarkers remains notably absent in clinical practice, primarily due to the limited supporting evidence in the literature. Biomarkers have demonstrated their transformative impact in various branches of medicine, including neurology and immunology, where they have facilitated early diagnosis, disease subtyping, treatment monitoring, prognosis assessment, and drug development.

However, efforts to distinguish between patients who will respond to treatment and those who will not have been unfruitful so far (25). Several studies have tried to identify the biological, psychopathological, and psychosocial correlates of depression, with particular attention to the dysfunction of the inflammatory system (26). Compared to patients with major depression who respond to pharmacological treatments, TRD patients have increased levels of proinflammatory cytokines, which indirectly reduce serotonin availability in the central nervous system (27) and the efficacy of antidepressant medications (28). Moreover, TRD is also associated with alterations in the hypothalamic-pituitary-adrenal (HPA) axis (29). A systematic review investigating the role of C-reactive protein (CRP) as a biomarker for MDD showed a low grade of inflammation was found in a percentage of MDD patients who were less responsive to treatment, suggesting that this could represent a subgroup of depressed patients with a different etiopathogenesis (30). Another studied biomarker is the brain-derived neurotrophic factor (BDNF), whose levels are significantly reduced in TRD patients compared to MDD, suggesting that the decreased levels of BDNF may be associated with biological resistance to traditional antidepressant treatments (31).

Taken together, available data suggest that chronic neuroinflammation might be implicated in the pathogenesis of MDD, with lower evidence about possible biomarkers of TRD (32). The identification of biomarkers of TRD holds relevant implications at clinical and research level. TRD biomarker could be used in clinical practice to identify in advance patients who are at higher risk to develop treatment resistance, facilitating the early detection of difficult to treat patients. Moreover, from a clinical perspective the availability of reliable biomarkers of TRD would be useful to assess a more precise prognosis of MDD patients, and to identify personalized and integrated treatments (which include psychotherapy and other psychosocial interventions) in order to reduce the risk of treatment resistance. At research level the identification of reliable biomarkers for TRD would be useful in order to develop new treatments strategies to be used in patients with TRD.

In this paper we review available studies assessing the full range of biomarkers compared to healthy controls in order to reshape TRD definition and improve its diagnosis, treatment, and prognosis.

## Methods

**Figure 1:**
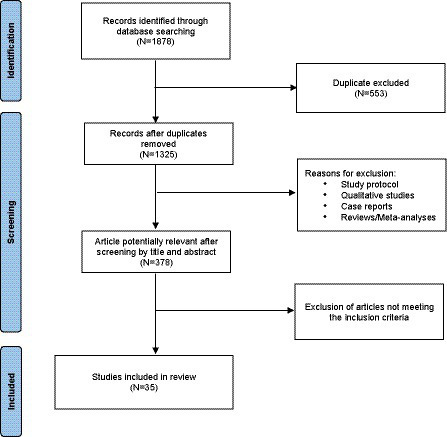
PRISMA flow diagram of selection of studies for inclusion in the review.

The keywords “treatment resistant depression” OR “TRD” matched with “biomarker,” “inflammation,” “hormone,” “cytokine” or “biological marker” were entered in the PubMed, ISI Web of Knowledge and SCOPUS databases for papers published from inception until April 6, 2023. Studies were included in the review if they: (1) included patients with a diagnosis of TRD; (2) assessed any biological marker for TRD; (3) included a control group of healthy subjects; (4) were written in English. Studies including other subsamples of patients (i.e., those with bipolar disorder) were included only if it was possible to extrapolate data on patients with unipolar TRD. We included only papers assessing biological markers in the review. Markers of different nature, such as those based on imaging, genetics and clinical evaluations were excluded from our analysis. Moreover, articles not providing a clear definition or utilizing ambiguous terminology for TRD were excluded. Only original articles were considered for the review. Additionally, the reference lists of all included papers were checked for the identification of other possible studies (Figure 1). The full reports of potentially relevant studies were obtained, and content of each paper was extracted.

For each paper, data on study design, sample characteristics, age range of recruited patients, biomarkers detected, psychopathological and psychosocial characteristics, TRD definition, and main results were independently extracted by four authors; discrepancies were resolved by discussion.

## Results

Entering the keywords in the relevant databases, 1,878 papers were identified; 553 were duplicates and excluded. 947 further papers were eliminated after reading the abstracts because they did not meet the inclusion criteria. After reading full-text papers, 212 more papers were excluded. Therefore, our review consists of 35 papers, grouped in the following five categories according to the main investigated biological correlates: studies on cytokines; other inflammatory markers; kynurenine; Brain-Derived Neurotrophic Factor (BDNF); and other clinical parameters (Table 1).

**Table 1:** Summary of studies included in the review.

Study and country	Sample size	Biomarker	Body fluid	TRD definition	Study design	Main results
Allen et al. (33), Ireland	35 TRD patients 20 HC	BDNF	Blood	Failure of two antidepressant trials	Cross-sectional	BDNF was lower in TRD patients compared to HC. sBDNF was significantly elevated only at 1 week following the first ketamine infusion in those classified as responders 1 week later. BDNF was not elevated following subsequent infusions
Bauer et al. (34), Brazil	36 TRD patients 31 HC	Salivary cortisol before and after DEX, phytohemagglutinin-induced T-cell proliferation, IL-2, TNFα, lymphocyte sensitivity to both cortisol and DEX	Saliva Blood	Failure of five different antidepressants trials	Cross-sectional	Basal morning cortisol levels from patients and controls did not differ nor did their T-cell proliferation and cytokine production. Ten out of 36 patients were classified as nonsuppressors and presented significantly higher post-DEX salivary cortisol levels than suppressors. Cells of nonsuppressors produced significantly less TNFα compared to suppressors. GC-induced suppression of lymphocyte proliferation and cytokine production were generally less marked in depressives compared with controls
Bauer et al. (35), Brazil	36 TRD patients 31 HC	Salivary cortisol and CD4+, CD8+, CD19+, CD56+, and HLADR+ cells distribution	Saliva Blood	Failure of five different antidepressants trials	Cross-sectional	No differences in basal salivary cortisol levels were found between patients and controls. Changes in cell redistribution (CD4+, CD8+, CD19+, CD56+, and HLADR+ cells) after DEX administration were more prominent in controls than in patients, but the effects of DEX varied dependent on whether patients exhibited DEX-induced suppression of cortisol secretion. Glucocorticoid-induced suppression of adhesion molecule expression was generally less marked in patients than controls
Carpenter et al. (36), USA	19 TRD patients 19 HC	Substance P	CSF	Failure to respond to at least two but not more than six antidepressant trials	Cross-sectional	Mean CSF substance P concentration was significantly lower in TRD patients on psychotropic medications than in the HC group
Cattaneo et al. (37), Italy	58 TRD patients 36 MMD responsive patients 36 MMD untreated patients 40 HC	IL-1-beta, IL-6, TNFα, MIF, glucocorticorticoid receptor, SGK1, FKBP5, P2RX7, CCL2, CXCL12, CRP, A2M, AQP4, ISG15, STAT1, and USP-18	Blood	Depressive symptoms (HDRS >13) while currently on an antidepressant at standard therapeutic dose for at least 6 weeks, plus at least one historical failure to a different antidepressant	Cross-sectional	Treatment-resistant and drug-free depressed patients had both increased inflammasome activation (higher P2RX7 and proinflammatory cytokines/chemokines mRNAs expression) and glucocorticoid resistance (lower GR and higher FKBP5 mRNAs expression), while responsive patients had an intermediate phenotype with lower CXCL12. Six mRNAs (P2RX7, IL-1-beta, IL-6, TNFα, CXCL12, and GR) distinguished treatment-resistant from responsive patients, even after adjusting for other variables that were different between groups
Chamberlain et al. (38), UK	102 TRD patients 48 Responsive MMD patients 48 Untreated MMD patients 54 HC	CRP	Blood	Patients with HDRS total score > 13; currently in treatment with a monoaminergic drug for at least 6 weeks	Cross-sectional	Compared with HC, CRP was significantly elevated in TRD, but was not in the treatment-responsive and untreated groups
Congio et al. (39), Brazil	24 TRD patients 82 HC	Leptin, CRP	Blood	HDRS-17 Total score > 16, after 8 to 12-weeks of several antidepressant trials	Cross-sectional	Higher levels of leptin, hs-CRP > 3 mg/L and higher BMI were found to be associated with TRD. The TRD patients with hs-CRP > 3 mg/L presented on average higher levels of leptin for the same BMI, compared to non-TRD
de Menezes Galvão et al. (40), Brazil	28 TRD patients 43 HC	Cortisol	Saliva Blood	Failure of two antidepressant trials	Placebo controlled trial	Baseline assessment showed blunted awakening salivary cortisol response and hypocortisolemia in patients, with TRD respect to HC
Galvão-Coelho et al. (41), Brazil	28 TRD patients 45 HC	CRP, IL-6, cortisol, BDNF, GOT, GPT	Blood	Failure of two antidepressant trials	Double blind placebo controlled-trial	Higher CRP levels and similar IL-6 levels in TRD patients compared to control group, adjusting for BMI. A significant inverse correlation between CRP and cortisol levels was found in patients. No correlation between CRP and BDNF, and between IL-6 and any variable in patient group. No correlation between CRP and IL-6 in the control group
Gur et al. (42), Israel	26 TRD patients 24 MDE (both MDD and BPD) patients 30 HC	AQP4-IgG	Blood	Failure of two antidepressant trials	Longitudinal	Absence of AQP4-IgG autoantibodies in all patients
Hoekstra et al. (43), Netherlands	20 TRD patients 29 HC	Biopterin, neopterin, phenylalanine, tyrosine, TRP, isoleucine, leucine, and valine	Blood	Failure to a prior treatment with a tricyclic antidepressant, lithium addition or an irreversible monoamine oxidase inhibitor	Longitudinal	Lower plasma biopterin concentration in TRD patients compared to HC. After treatment, biopterin increased in TRD patients with psychotic features. The plasma phenylalanine/tyrosine ratio normalized after ECT. Mean tryptophan concentration was lower in TRD than in HC
Huang et al. (44), Taiwan	20 TRD patients 14 responsive MDD patients 34 HC	CPR, sIL-2R, sIL-6R, TNFα-R1	Blood	Failure of two antidepressant trials	Cross-sectional	MDD patients had higher serum concentrations of TNFα R1. Higher serum concentrations of TNFα R1 in TRD patients than in healthy controls or non-TRD group. The most significant finding from this study was the correlation of increased serum concentrations of TNFα R1 and impaired glutamatergic neurotransmission in the caudate nucleus and anterior cingulate cortex in patients with TRD
Juruena et al. (45), UK	12 TRD patients 12 HC	Cortisol	Saliva	Failure of two antidepressant trials	Cross-sectional	Higher salivary cortisol levels in TRD patients compared with controls after all challenges. In these patients the provision of spironolactone did not increase cortisol compared to placebo; spironolactone with prednisolone had no effect on the suppressive effects of prednisolone. Patients with TRD had a reduction in the conversation of spironolactone to the active metabolite canrenone
Lauden et al. (46), Israel	570 TRD patients 2,850 MDD patients 2,850 HC	WBC, lymphocytes, eosinophils, basophils, platelets, MPV, glucose, TSH, CRP, ESR, C3, C4, antinuclear antibodies, RF, IgE	Blood	Presence of minimal improvement or no improvement with at least two different classes of antidepressants, at adequate doses and durations (at least 6 weeks)	Cross-sectional	Higher levels of blood WBC, lymphocytes, platelets, C-reactive protein, ESR, C3 and C4 levels in TRD patients compared controls
Maes et al. (47), Belgium	28 TRD patients 8 responsive MDD patients 15 HC	DPP IV	Blood	Failure of two antidepressant trials	Cross-sectional	Significantly lower derum DPP IV activity in major depressed subjects, irrespective of treatment resistance, than in normal volunteers; subchronic treatment with antidepressants has no significant effect on serum DPP IV activity; serum DPP IV is related to immune- as well as inflammatory markers of major depression
Maes et al. (48), Belgium	23 TRD patients 9 responsive MDD patients 15 HC	Zn and Cu	Blood	Failure of two antidepressant trials	Longitudinal	Decreased Serum Zn levels in TRD patients; treatment with antidepressants does not alter the initially lower Zn levels, although antidepressant treatment significantly reduces serum Cu levels; lower serum Zn is significantly related to immune/inflammatory markers
Maes et al. (49), Belgium	28 TRD patients 7 MDD patients 15 HC	IL-6, IL-6R, IL-1Ra, sCD8, CC16, and Zn	Blood	Treatment resistance according to Thase and Rush criteria	Cross-sectional	Significantly higher serum IL-6 levels in TRD subjects, while there were no significant differences between normal volunteers and non-TRD patients, and between patients with and without TRD
Maes et al. (50), Belgium	19 TRD patients 16 responsive MDD patients 22 HC	CoQ10	Blood	Presence of (a) failure of two antidepressant trials; (b) failure to respond to augmentation treatment; (c) point b plus failure to respond to two augmentation strategies; (d) previous stage plus non-response to ECT	Cross-sectional	Plasma CoQ10 was significantly lower in patients with TRD and with Chronic Fatigue Syndrome than in the other depressed patients. No significant correlation between plasma CoQ10 and the HDRS
Markopoulou et al. (51), UK	28 TRD patients 40 HC	DHEA, cortisol	Blood	Failure of two antidepressant trials. Degree of resistance was staged according to the Thase and Rush criteria	Observational	Cortisol levels were significantly higher in patients than controls, but DHEA levels did not differ. The ratio of cortisol/DHEA was significantly elevated in patients
Nasca et al. (52), USA	11 TRD patients 26 MD patients 26 HC	LAC	Blood	History of nonresponse to at least two antidepressant trials	Cross-sectional	Compared to HC, decrease in LAC was larger in TRD patients, among whom childhood trauma and, specifically, a history of emotional neglect and being female, predicted the decreased LAC
Pisoni et al. (53), United Kingdom	36 TRD patients 36 HC	Tie2, BDNF, VEGF, VEGFC, VEGFD, PlGF, bFGF, and sFlt1	Blood	Score > 7.5 using the Maudsley Staging Method	Longitudinal	Deficit of peripheral growth factors in TRD patients. Higher Tie2 levels in TRD patients than controls, while lower VEGFC and BDNF levels in TRD participants. Levels of VEGF were not significantly different between patients and controls A decrease of VEGF 260 and VEGFC over time in TRD patients was reported. No changes were seen in levels of BDNF following antidepressant treatment. TRD patients showed significantly lower levels of VEGFD at admission compared to responders
Rengasamy et al. (54), USA	103 TRD patients 43 HC	IL-6	Blood CSF	Failure of three antidepressant trials	Cross-sectional	Higher levels of plasma IL-6 were found in TRD compared to HC
Sanchez-Carro et al. (55), Spain	59 TRD patients 32 MDD patients 80 HC	TNFα and CRP	Blood	Failure of two antidepressant trials, or non-response to the augmentation treatments	Cross-sectional	TNFα and CRP were relevant for the differentiation of the group of patients from the HC group
Sasaki et al. (56), Japan	10 TRD patients 27 MDD patients 25 HC	OXT	Blood	Failure of two antidepressant trials and not responding to at least eight sessions of cognitive behavioral therapy	Cross-sectional	Serum OXT levels in TRD patients were higher compared to HC
Schwieler et al. (57), Sweden	19 TRD patients 22 HC	IL-1β, IL-2, IL-6, IL-8, IL-10, IL-12p70, TNFα, IFN-γ, GM-CSF, KYNA, and QUIN	Blood	Patients had been adequately treated with oral antidepressant, but had not responded	Observational	Increased plasma levels of IL-6 in TRD patients compared HC. Decreased plasma levels of KYNA and significantly increased QUIN/KYNA ratio in TRD Plasma levels of tryptophan, kynurenine, and QUIN did not differ between patients and controls. There was a significant inverse correlation between symptom severity and kynurenine levels at baseline
Sowa-Kucma et al. (58), Poland	42 TRD patients 72 responsive MDD patients 50 HC	IL-1α, IL-1RA, IL-2R, IL-6R, sTNF-R1, sTNF-R2, TBARS	Blood	Failure of two antidepressant trials	Cross-sectional	TRD is characterized by increased sIL-6R levels as compared with controls and depressed patients without TRD, lowered sTNF-R2 levels as compared to non-TRD patients and increased TBARS levels as compared with all other study samples
Strawbridge et al. (59), UK	129 TRD patients 28 HC	IL-6, CRP, TNFα, and IL-10	Blood	Non-responsive to at least two antidepressants	Two-arm parallel-group, double-blind, randomized, placebo-controlled trial	CRP, TNFα and IL-6 were elevated in TRD patients compared to HC. Other inflammatory proteins did not mediate or moderate treatment outcomes
Strawbridge et al. (60), UK	36 TRD patients 36 HC	CRP, IFNα, IFNγ, IL-10, IL-12, IL-12p70, IL-13, IL-15, IL-16, IL-17, IL-1α, IL1β, IL-2, IL-4, IL-5, IL-6, IL-7, IL-8 (CXCL8), TNFα, TNFβ, Eotaxin (CCL11), Eotaxin-3 (CCL26), GM-CSF, IP-10 (CXCL10), MCP1 (CCL2), MCP4 (CCL13), Mip1a (CCL3), Mip1b (CCL4), SAA, sICAM1 (sCD54), sVCAM1 (sCD106), and TARC (CCL17)	Blood	TRD was assessed using the Maudsley Staging Method staging tool	Longitudinal	Patients with TRD reported higher proteomic inflammatory activity than HC; elevated inflammation is predictive of a more severe or resistant depressive illness both retrospectively (i.e., prior to inpatient treatment, in the current episode) and prospectively (predicting more severe depressive symptoms in the months after discharge)
Szałach et al. (61), Poland	20 TRD patients 13 HC	CD28, CD69, CD25, CD95, HLA-DR, IL12p70, TNFα, IL-10, IL-6, IL-1β, and IL-8	Blood	Failure of two antidepressant trials	Cross-sectional	Lower percentage of CD3 + CD4 + CD25+ and CD3 + CD8 + CD95+ cells in TRD patients than HC, lower serum levels of IL-12p70 and TNFα, and significantly higher IL-8 levels
Uint et al. (62), Brasil	34 TRD patients 43 BPD patients 41 HC	TNFα, IL-1β, IL-6, BDNF, and CRP	Blood	Failure of two antidepressant trials	Cross-sectional	BDNF and IL-1β plasma concentrations were increased in TRD compared to HC
Vandoolaeghe et al. (63), Belgium	27 TRD patients 9 responsive MDD patients 15 HC	TSH, T4	Blood	Failure of two antidepressant trials	Cross-sectional	No significant differences in basal TSH or T4 in TRD was found
Van Hunsel et al. (64), Belgium	29 TRD patients 8 responsive MDD patients 29 HC	TSP, albumin, alpha1, alpha2, beta, and gamma-globulin	Blood	Failure of two antidepressant trials	Longitudinal	Significantly lower TSP and percentage and concentration of serum albumin (Alb) and y-globulin fraction in TRD than in HC Serum beta-globulin concentrations were significantly lower in TRD subjects than in HC
Wu et al. (65) China	30 TRD patients 30 responsive MDD patients 30 HC	Cortisol, nesfatin-1, CRP, TNFα, IL-6, 1 L-1β	Blood	Ineffective treatments for 3 months with two or more different antidepressants in sufficient quantity	Cross-sectional	Serum cortisol, CRP, TNFα, and IL-6 levels were significantly higher in TRD than in HC. Serum nesfatin-1 levels in the non-TRD group were significantly lower than HC and TRD groups, and significantly higher serum IL-1β levels in the non-TRD group than in the control and TRD groups
Zhou et al. (66), China	68 TRD patients 6 HC	TRP, KYN, and KYNA	Blood	Failure of two antidepressant trials	Longitudinal	Lower serum levels of TRP and KYNA and the KYNA/KYN ratio and higher KYN/TRP ratio in TRD patients than in HC
Zincir et al. (67), Turkey	50 TRD patients 30 HC	IL-1, IL-6, TNFα, IL-10, IL-4, and IFN-gamma	Blood	Failure of two antidepressant trials	Prospective, non-randomized, controlled study	Higher levels of IL-1, TNFα, and IL-10 before treatment in TRD than in HC. No significant difference in the levels of IL-6 before and after treatment when compared to the control group

AQP4, aquaporin-4; A2M, alpha-2-macroglobulin; BDNF, brain-derived neutrophic factor; bFGF, basic fibroblast growth factor; BPD, bipolar disorder; CCL, CC motif chemokine ligand; CD, cluster of differentiation; CoQ10, Q10 Coenzyme; CRP, C-reactive protein; CSF, cerebrospinal fluid; Cu, cupper; CXCL, CXC motif chemokine ligand; C3, complement component 3; C4, complement component 4; CC16, clara cell protein; DDP IV, dipeptidyl peptidase 4; Dex, dexamethasone; DHEA, dehydroepiandrosterone; ECT, elettroconvulsive therapy; ERS, erythrocyte sedimentation rate; FKBP5, FK506 binding protein 5L GM-CSF, granulocyte-macrophage colony-stimulating factor; GOT, glutamic oxaloacetic transaminase; GPT, glutamic pyruvic transaminase; HC, healthy control; HDRS, Hamilton Depression Rating Scale; HLA, human leukocyte antigen; Ig, immunoglobulin; IL, interleukin; IL1RA, IL-1 receptor antagonist; IL-2R, IL-2 receptor; INF, interferon; KYN, kynurenin; KYNA, kynurenic acid; LAC, acetyl-L-carnitine; Mcp, monocyte chemoattractant protein-1; MDD, major depressive disorder; MDE, major depressive episode; MIF, macrophage migration inhibitory factor; Mip, macrophage inflammatory protein; MPV, medium platelet volume; OXT, oxytocin; PlGF, placental growth factor; P2RX7, purinergic receptor; QUIN, quinolinic acid; RF, reumatoid factor; sFlt1, soluble fms-like tyrosine kinase-1 (VEGF receptor-1); sICAM, soluble intercellular adhesion molecules; sVCAM, soluble vascular cell adhesion molecule; sIL2R, soluble IL-2 receptor; sIL6R, soluble IL-2 receptor; sTNF-R1, soluble TNF-receptor1; sTNF-R2, soluble TNF-receptor2; TARC, thymus- and activation-regulated chemokine; TBARS, thiobarbituric acid reactive substances; Tie-2, angiopoietin-1 receptor; TNF, tumor necrosis factor; TNFαR1, tumor necrosis factor-alpha receptor subtype 1; TRD, treatment-resistant depression; TSH, thyroid stimulating hormone; TSP, total serum protein; T3, triiodothyronine; T4, thyroxine; VEGF, Vascular endothelial growth factor; WBC, white blood cells; Zn, Zinc.

### Cytokines

With respect to IL-1, available data are still inconsistent. In fact, while Uint et al. (62) found higher IL-1b plasma levels in TRD compared to HC, Zincir et al. (67) and Wu et al. (65) found lower IL-1b levels of in TRD patients.

All available studies found increased plasma levels of IL-6 in TRD patients compared to HC (28, 49, 54, 65).

Seven studies addressed the correlation between TNF-α and TRD. Sanchez-Carro et al. (55) provided data supporting the role of TNF-α in discriminating between TRD and HC using a machine learning approach. These findings were replicated in a case–control cross-sectional study on elderly TRD patients, where TNF-α levels were significantly higher in TRD than in the HC group (65). In addition, in a double-blind, randomized, placebo-controlled trial, Strawbridge et al. (59) found that the baseline pro-inflammatory proteins, including TNF-α, were significantly higher in TRD patients than in HC, after controlling for gender, age, childhood adversity and BMI. On the other hand, one study found no difference in the production of lipopolysaccharide induced-TNF-α in peripheral blood mononuclear cells (34), while other reports (61, 67) found decreased TNF-α levels in TRD compared to HC. Interestingly, one study reported higher serum concentrations of TNF-α receptor subtype 1 (TNF-α R1) titers in TRD patients compared to HC (44).

In a randomized controlled trial, Zincir et al. (67) found higher levels of IL-10 in TRD compared to HC, while another study found no difference between TRD patients and healthy controls (59).

Other cytokines which have been explored as potential biomarkers of TRD include IL-12, IL-5, Interferon gamma (IFN-gamma), IL-8 and IL-4. Szałach et al. (61) reported lower levels of serum IL-12 and higher levels of IL-8 in TRD patients vs. HC. Strawbridge et al. (60) found higher levels of IL-8 in TRD patients compared to controls, associated with elevated titers of IL-5. Moreover, IL-4 blood levels were significantly higher in TRD than in the control group (67), while no difference in phytohemagglutinin (PHA)-induced IL-2 production has been found between patients and controls (34). One study found higher IFN-gamma titers in TRD than in the control group (67).

### Other inflammatory markers

Despite consolidated evidence on cortisol levels in MDD, only a few studies have been carried out in patients with TRD. Markopoulou et al. (51) and Wu et al. (65) found higher cortisol serum levels in TRD vs. HC. Interestingly, Juruena et al. (45) found an impaired activity of glucocorticoid receptors (GRs) in TRD group compared to HC. de Menezes Galvão et al. (40) carried out a RCT on the effect of ayahuasca on the hypothalamic-pituitary-adrenal axis (HPA) and found that at baseline TRD patients exhibit blunted awakening salivary cortisol response and hypocortisolemia compared to HC.

Four studies (38, 39, 41, 59) found elevated levels of C-reactive protein (CRP) in TRD patients compared to HC, two studies reported no differences between cases and controls (44, 62), while Sanchez-Carro et al. (55) found that CRP does not discriminate between the two groups.

Four studies investigated immunological cells populations in TRD patients compared to HC. In particular, two studies found no differences in lymphocyte proliferation (34) and central populations of T cells between TRD patients and HC (61). However, in a large trial by Lauden et al. (46) on 570 TRD patients and 2,850 HC, higher levels of blood WBC, lymphocytes and platelets were found in the TRD group. Another study on lymphocyte sensitivity to dexamethasone (DEX) intake found that changes in cell redistribution after DEX administration were more prominent in TRD patients than in controls, but the effects of DEX were dependent on DEX-induced suppression of cortisol secretion (35).

### Kynurenine

We found three studies on the kynurenine pathway in TRD. Zhou et al. (66) found lower serum concentrations of tryptophan (TRP), kynurenic acid (KYNA) and the KYNA/kynurenine (KYN) ratio, and a higher KYN/TRP ratio in TRD patients compared to HC. Also, Schwieler et al. (57) found an altered kynurenine metabolism in TRD patients, in particular decreased plasma levels of KYNA and significantly increased quinolinic acid/kynurenine ratio. However, one study found no difference between TRD and HC in the plasma levels of tryptophan, KYNA, and quinolinic acid (QUIN).

### Brain-Derived Neurotrophic Factor

Four studies have explored the role of BDNF in TRD. In a randomized double-blinded placebo-controlled trial using a parallel-arm design of ayahuasca vs. placebo, no correlation was found between plasma levels of BDNF and TRD (41). Two studies reported lower levels of BDNF in TRD compared to HC (33, 53), while Uint et al. (62) found opposite results.

### Other hematological parameters

Several other hematological parameters have been investigated in TRD patients. In particular, lower serum albumin levels were found in TRD patients compared to controls (64), while no significant difference in the levels of basal Thyroid Stimulating Hormone (TSH) and T4 were detected between major depressed patients with or without TRD and non-TRD (46, 63). One study showed lower vascular endothelial growth factor (VEGF) titers in TRD patients compared to HC (53). One study found reduced baseline levels of enzyme cofactor biopterin (involved in the synthesis of neurotransmitters like serotonin, dopamine, and norepinephrine) in TRD patients compared to HC (43). Significantly decreased serum levels of acetylating molecule acetyl-L-carnitine (LAC) were observed in TRD patients compared to HC (52). Another study reported higher serum levels of oxytocin (OXT) in a sample of adolescents with TRD compared to age-matched HC (56).

Gur et al. (42) found that TRD patients are more frequently seronegative to Aquaporin-4 (an astrocyte water channel protein) autoantibodies (AQP4-IgG) compared to HC. However, another study reported no statistical difference in the expression of AQP4 gene between TRD and HC (37).

Interestingly, two studies assessed zinc (Zn) serum levels: Maes et al. (48) found significantly lower levels of serum Zn in TRD than in HC, which were inversely correlated with IL-6 titers (49). The same authors showed a significantly lower serum activity of dipeptidyl peptidase IV (DPP IV), a serine protease with a role in cytokine production, in TRD than in HC (47), and significantly lower levels of the antioxidant Coenzyme Q10 compared to responsive-MDD patients (50). Sanchez-Carro et al. (55) reported that glutathione and 4-hydroxynonenal (HNE) could serve as variables to discriminate between TRD patients and HC. Moreover, one study investigated the role of stress-related neuropetide Substance P (SP) in the central nervous system (CNS), by means of standard lumbar puncture techniques (36). Authors reported that TRD patients taking psychotropic medications had significantly lower mean cerebrospinal fluid SP concentration than HC (53).

## Discussion

The underlying biological mechanisms that contribute to development and maintenance of TRD are not yet elucidated. The identification of reliable biomarkers would allow an early identification, proper diagnosis and treatment of TRD, improving the chance of a successful outcome (68). However, only a small number of biological alterations seem to correlate with TRD, in particular some cytokines, the kynurenine pathway catabolites, CRP, BDNF and cortisol.

The role of inflammation, and in particular of cytokines, in the pathophysiology of mental disorders has been recently highlighted (69), following a new wave of studies using modern biological techniques (70, 71). While several evidence shows an involvement of the immunological systems in MDD, suggesting that the communication between immune and brain systems might be mediated by increased cytokine levels (72, 73), only a limited number of studies investigating the role of inflammation and of cytokine alteration in TRD have been found, despite the presence of low-grade neuroinflammation has been reported to be more frequently in patient with treatment resistant major depression, rather than in responders and healthy controls (74, 75).

Available evidence has reported that TNF-α, whose blood concentration has shown a significant improvement after treatments with antidepressants, is the most relevant biomarker to discriminate TRD patients from both to HC and to treatment-responsive MDD patients (55, 74). In the Central Nervous System (CNS) TNF-α promotes serotonin metabolism and enhances the serotonin transporter’s activity (76). In particular, reduced levels of TNF-α could be associated to a reduced activity of serotonin transporter, thus influencing the effectiveness antidepressants, like selective serotonin reuptake inhibitors (SSRIs) (76). Consequently, the assessment of TNF-α levels could have potential clinical relevance for TRD patients who have experienced several unsuccessful trials of antidepressant treatments (77).

Two studies found increased levels of IL-8 in TRD patients compared to healthy controls. IL-8 is produced by monocytes, macrophages, and neutrophils and exerts a pro-inflammatory action, by facilitating neutrophil migration. It is also synthetized in SNC by microglia can synthesize IL-8 in response to proinflammatory stimuli; it has also been reported that anti-inflammatory cytokines can downregulate its production and release in the SNC (20). IL-8 levels have been found to be consistently elevated in TRD patients also when they are compared to MDD responsive individuals, suggesting that this cytokine could be a potential biomarker for TRD. However, this hypothesis needs to be confirmed by further larger longitudinal studies, with standardized diagnostic criteria and treatment-specific analyzes. Additionally, a more comprehensive understanding of the role of IL-8 in TRD might come from multi-modal research approaches, integrating genetic, imaging, and clinical data. Reviewed studies are insufficient to draw any other firm consideration about the role of the other cytokines, such as IL-2, IL-5, and IL-12, in TRD pathophysiology.

The BDNF has also been assessed as a biomarker in the pathophysiology of TRD. The BDNF belongs to the family of neurotrophins, a group of growth factors that support the survival, development, and function of neurons in the brain and peripheral nervous system (78). Inflammation, which is associated with increased cytokines production, affects BDNF expression, although the exact biological pathway is not fully elucidated (79). Chronic stress induces a reduction in BDNF concentration (80), but studies analyzing serum BDNF levels in TRD conveyed conflicting results (81). In fact, while some studies reported a reduction of BDNF concentration (53, 64), others found an increase of BDNF levels (62) or no difference between TRD and healthy controls. The inconsistency of these results might be due to the fact that serum analysis of BDNF concentrations is variable and scarcely reliable, unless Polymerase Chain Reaction (PCR) is used.

Many studies reported increased cortisol levels in TRD patients (51, 65), suggesting an alteration in HPA axis. One hypothesis regarding cortisol modulation in depression indicates a form of HPA axis fatigue with an underlying hypocortisolism both in salivary and plasma samples (34, 40). In fact, chronic low levels of cortisol can cause weakness, loss of appetite and immunological dysfunctions, which are symptoms commonly associated to depression (82, 83). However, the inconsistency of results reported in studies included in the present review can be explained by the fact that antidepressant treatments can alter HPA axis functions. Therefore, in order to fully understand the role of cortisol in depression, studies comparing medicated vs. non medicated patients are needed (84).

Several studies found alterations in the number of blood immune cells. Evidence shows that TRD patients can have increased leucocytes and possibly platelets; however, the role of immune cells in TRD should be better investigated. In fact, studies including a higher number of participants reported an increase in immunological cells, such as neutrophils and platelets in TRD patients vs. healthy controls; however, these differences were not statistically significant when comparing MMD and TRD, challenging the view that they can represent different pathologies along the affective spectrum (46).

In the present review, an alteration in the kynurenine pathway (KP) has been reported in several studies. This result is of particular relevance, since the vast majority (~95%) of tryptophan (TRP) is metabolized via KP in kynurenine (KYN), quinolinic acid (QUIN) and kynuretic acid (KYNA), while only a small part of TRR is used to synthetize monoamines, implicated in the pathophysiology of MDD, including noradrenaline and serotonin (85). Enzymes of the KP, can be activated by pro-inflammatory cytokines, which may lead to TRP depletion (86). Results of the present review confirm this hypothesis, despite they need to be replicated in larger samples.

Treatment-resistant depression represents a significant challenge in mental health care, making a priority the need to identify the etiological pathways of this complex mental disorder. Numerous additional biological pathways, including biopterin, acetyl-L-carnitine, oxytocin, zinc, glutathione, nesfatin-1, and dipeptidyl peptidase IV, have been investigated in TRD. In particular, biopterin, a critical cofactor in neurotransmitter synthesis, has shown potential relevance in TRD (87). Alterations in biopterin metabolism have been associated with the dysregulation of serotonin, dopamine, and norepinephrine systems, all of them being implicated in depression (88). Similarly, Acetyl-L-carnitine, an endogenous compound involved in cellular energy metabolism and neuroprotection, has demonstrated antidepressant effects in clinical studies, indicating its potential as a therapeutic target for TRD (89). While the studies on pathways of biopterin and acetyl-L-carnitine seem promising to enhance our understanding of major depression and of TRD, others - including aquaporin-4, vascular endothelial growth factor (VEGF), and thyroid-stimulating hormone (TSH) - have yield fewer compelling results. However, the current level of evidence for these pathways is still low, and any consideration about the potential role in TRD remains speculative.

The existing literature on the biological correlates of TRD is explored by numerous studies, but the comparability of their findings and methods often proves challenging mainly due to methodological disparities and clinical characterization differences. These variations encompass the utilization of diverse laboratory techniques and the incorporation of inclusion criteria grounded in distinct conceptual definitions. As a consequence, the synthesis of this body of research faces obstacles in drawing definitive conclusions about the underlying biological mechanisms of TRD.

In the analysis of the selected articles conflicting outcomes have emerged. Nevertheless, certain cytokines, such as IL-6 and TNF-α, have demonstrated a more extensive body of supporting evidence. A significant proportion of the examined cytokines, however, lacked a sufficient number of studies for meaningful cross-comparisons, rendering the available evidence insufficient to derive preliminary conclusions. Moreover, notwithstanding the presence of evidentiary support in other domains of psychiatric pathologies, the cortisol pathway exhibited incongruent findings in the context of TRD. Additionally, the available data regarding BDNF appear challenging to compare due to methodological disparities in the analysis, which may account for the incongruity of the results.

This review is subject to several limitations, that are hereby acknowledged. First and foremost, a significant challenge in our synthesis of findings is the inconsistency in the definition of treatment-resistant depression across studies. The lack of a standardized and universally accepted definition hampers the possibility to draw definitive conclusions regarding biomarkers associated with this specific depressive phenotype. Additionally, methodological limitations within included studies, such as variations in sample collection and processing techniques, assay methodologies, and data analysis approaches, introduce potential sources of bias, reducing the comparability and generalizability of results. Another common limitation observed in available studies is represented by the relatively small sample sizes, which may limit the statistical power of studies. Therefore, caution is needed when interpreting the findings of this review, and further well-designed studies with larger and more homogeneous samples are warranted to overcome these limitations and provide more robust evidence regarding biomarkers of TRD.

In conclusion, although the notion of TRD lacks coherence and standardization (90, 91), some evidence suggests a biological alteration in TRD. However, the future perspectives for research on the biological correlates of TRD are both promising and challenging (92). To advance our understanding of TRD’s biological underpinnings, it is imperative to establish a more robust conceptual framework for TRD, which include the resistance to psychotherapeutic interventions, also. Additionally, future studies should aim to include well-characterized, medication-naïve patient samples and adopt longitudinal designs to assess biomarker variations over time. Based on the findings of this review, it becomes evident that prioritizing the analysis of biomarker panels, rather that single biomarkers, is imperative. Finding a biosignature of TRD, coming from a panel of biomarkers, not only enables a more comprehensive understanding of biological processes underlying mental disorder but also offers an opportunity to develop targeted treatments able to influence it and to modify the long-term outcome of TRD. Lastly, future studies should include strategies to identify patient with pseudoresistance to pharmacological treatments (23), due to poor compliance to pharmacological treatments.

## Author contributions

EM: Conceptualization, Methodology, Writing – original draft, Writing – review & editing. GS: Conceptualization, Methodology, Writing – original draft, Writing – review & editing, Supervision. AB: Writing – review & editing, Data curation. BD: Data curation, Writing – review & editing. MD: Data curation, Writing – review & editing. MVL: Methodology, Writing – review & editing. FM: Methodology, Writing – review & editing. FL: Data curation, Writing – review & editing. ML: Conceptualization, Methodology, Writing – original draft, Writing – review & editing.

## References

[1] Boschloo L Hieronymus F Cuijpers P ICECA Work Group. Clinical response to SSRIs relative to cognitive behavioral therapy in depression: a symptom-specific approach. World Psychiatry. (2022) 21:152–3. doi: 10.1002/wps.20944, PMID: 35015348 PMC8751549

[2] World Health Organization. Depression (2021). Available at: https://www.who.int/news-room/fact-sheets/detail/depression (Accessed April 25, 2023).

[3] Miguel C Karyotaki E Cuijpers P Cristea IA. Selective outcome reporting and the effectiveness of psychotherapies for depression. World Psychiatry. (2021) 20:444–5. doi: 10.1002/wps.20900, PMID: 34505363 PMC8429345

[4] Trivedi MH. Major depressive disorder: remission of associated symptoms. J Clin Psychiatry. (2006) 67:27–32. 16848674

[5] Greenberg PE Fournier AA Sisitsky T Simes M Berman R Koenigsberg SH. The economic burden of adults with major depressive disorder in the United States (2010 and 2018). PharmacoEconomics. (2021) 39:653–65. doi: 10.1007/s40273-021-01019-4, PMID: 33950419 PMC8097130

[6] Steger MF. Meaning in life is a fundamental protective factor in the context of psychopathology. World Psychiatry. (2022) 21:389–90. doi: 10.1002/wps.20916, PMID: 36073697 PMC9453886

[7] Furukawa TA Shinohara K Sahker E Karyotaki E Miguel C Ciharova M. Initial treatment choices to achieve sustained response in major depression: a systematic review and network meta-analysis. World Psychiatry (2021) 20:387–96. doi: 10.1002/wps.20906, PMID: 34505365 PMC8429344

[8] Murphy JA Sarris J Byrne GJ. A review of the conceptualisation and risk factors associated with treatment-resistant depression. Depress Res Treat. (2017) 2017:4176825–10. doi: 10.1155/2017/4176825, PMID: 28840042 PMC5559917

[9] Ban TA. Prolegomenon to the clinical prerequisite: psychopharmacology and the classification of mental disorders. Prog Neuro-Psychopharmacol Biol Psychiatry. (1987) 11:527–80. doi: 10.1016/0278-5846(87)90019-4 2892227

[10] Thase ME Rush AJ. When at first you don’t succeed: sequential strategies for antidepressant nonresponders. J Clin Psychiatry. (1997) 58:23–9. 9402916

[11] Ventriglio A Bhugra D Sampogna G Luciano M De Berardis D Sani G. From dysthymia to treatment-resistant depression: evolution of a psychopathological construct. Int Rev Psychiatry. (2020) 32:471–6. doi: 10.1080/09540261.2020.1765517, PMID: 32436408

[12] Cuijpers P Quero S Noma H Ciharova M Miguel C Karyotaki E. Psychotherapies for depression: a network meta-analysis covering efficacy, acceptability and long-term outcomes of all main treatment types. World Psychiatry (2021) 20:283–93. doi: 10.1002/wps.20860, PMID: 34002502 PMC8129869

[13] European Medicines Agency. Guideline on clinical investigation of medicinal products for the treatment of depression. (2013). Available at: https://www.ema.europa.eu/en/news/european-medicines-agency-publishes-guideline-clinical-investigation-medicines-depression (Accessed April 25, 2023).

[14] Fekadu A Wooderson S Donaldson C Markopoulou K Masterson B Poon L. A multidimensional tool to quantify treatment resistance in depression: the Maudsley staging method. J Clin Psychiatry. (2009) 70:177–84. doi: 10.4088/jcp.08m04309 19192471

[15] McIntyre RS Filteau MJ Martin L Patry S Carvalho A Cha DS. Treatment-resistant depression: definitions, review of the evidence, and algorithmic approach. J Affect Disord. (2014) 156:1–7. doi: 10.1016/j.jad.2013.10.043, PMID: 24314926

[16] Rush AJ Sackeim HA Conway CR Bunker MT Hollon SD Demyttenaere K. Clinical research challenges posed by difficult-to-treat depression. Psychol Med. (2022) 52:419–32. doi: 10.1017/S0033291721004943, PMID: 34991768 PMC8883824

[17] Stein DJ Shoptaw SJ Vigo DV Lund C Cuijpers P Bantjes J. Psychiatric diagnosis and treatment in the 21st century: paradigm shifts versus incremental integration. World Psychiatry. (2022) 21:393–414. doi: 10.1002/wps.20998, PMID: 36073709 PMC9453916

[18] Anderson IM. We all know what we mean by treatment-resistant depression - don't we? Br J Psychiatry. (2018) 212:259–61. doi: 10.1192/bjp.2018.56 29693539

[19] Meng R Yu C Liu N He M Lv J Guo Y. Association of Depression with all-Cause and Cardiovascular Disease Mortality among Adults in China. JAMA Netw Open. (2020) 3:e1921043. doi: 10.1001/jamanetworkopen.2019.21043, PMID: 32049295 PMC7212017

[20] Kim H Turiano NA Forbes MK Kotov R Krueger RF Eaton NR. Internalizing psychopathology and all-cause mortality: a comparison of transdiagnostic vs. diagnosis-based risk prediction. World Psychiatry. (2021) 20:276–82. doi: 10.1002/wps.20859, PMID: 34002512 PMC8129836

[21] Bergfeld IO Mantione M Figee M Schuurman PR Lok A Denys D. Treatment-resistant depression and suicidality. J Affect Disord. (2018) 235:362–7. doi: 10.1016/j.jad.2018.04.016 29665520

[22] Möller HJ Seemüller F Schennach R Gupta RK. Treatment-resistant depression: a separate disorder – a new approach In: Kasper S Montgomery S, editors. Treatment-resistant depression. London: Willey Blackwell (2013). 21–41. doi: 10.1002/9781118556719.ch2

[23] Leichsenring F Steinert C Rabung S Ioannidis JPA. The efficacy of psychotherapies and pharmacotherapies for mental disorders in adults: an umbrella review and meta-analytic evaluation of recent meta-analyses. World Psychiatry. (2022) 21:133–45. doi: 10.1002/wps.20941, PMID: 35015359 PMC8751557

[24] FDA-NIH Biomarker Working Group. BEST (biomarkers, EndpointS, and other tools) resource. Silver spring (MD): Food and Drug Administration (US); Bethesda (MD): National Institutes of Health (US). (2006). Available at: www.ncbi.nlm.nih.gov/books/NBK326791/. 27010052

[25] Owen MJ Williams NM. Explaining the missing heritability of psychiatric disorders. World Psychiatry. (2021) 20:294–5. doi: 10.1002/wps.20870, PMID: 34002520 PMC8129850

[26] Fisher AJ Song J Soyster PD. Toward a systems-based approach to understanding the role of the sympathetic nervous system in depression. World Psychiatry. (2021) 20:295–6. doi: 10.1002/wps.20872, PMID: 34002517 PMC8129862

[27] Miller AH Maletic V Raison CL. Inflammation and its discontents: the role of cytokines in the pathophysiology of major depression. Biol Psychiatry. (2009) 65:732–41. doi: 10.1016/j.biopsych.2008.11.029, PMID: 19150053 PMC2680424

[28] Liu JJ Wei YB Strawbridge R Bao Y Chang S Shi L. Peripheral cytokine levels and response to antidepressant treatment in depression: a systematic review and meta-analysis. Mol Psychiatry. (2020) 25:339–50. doi: 10.1038/s41380-019-0474-5, PMID: 31427752

[29] Markopoulou K Fischer S Papadopoulos A Poon L Rane LJ Fekadu A. Comparison of hypothalamo-pituitary-adrenal function in treatment resistant unipolar and bipolar depression. Transl Psychiatry. (2021) 11:1–8. doi: 10.1038/s41398-021-01343-5 33903590 PMC8076168

[30] Orsolini L Pompili S Tempia Valenta S Salvi V Volpe U. C-reactive protein as a biomarker for major depressive disorder? Int J Mol Sci. (2022) 23:1616. doi: 10.3390/ijms23031616, PMID: 35163538 PMC8836046

[31] Watson D Levin-Aspenson HF Waszczuk MA Conway CC Dalgleish T Dretsch MN. Validity and utility of hierarchical taxonomy of psychopathology (HiTOP): III. Emotional dysfunction superspectrum. World Psychiatry. (2022) 21:26–54. doi: 10.1002/wps.20943, PMID: 35015357 PMC8751579

[32] Marrie RA Bernstein CN. Psychiatric comorbidity in immune-mediated inflammatory diseases. World Psychiatry. (2021) 20:298–9. doi: 10.1002/wps.20873, PMID: 34002519 PMC8129838

[33] Allen AP Naughton M Dowling J Walsh A Ismail F Shorten G. Serum BDNF as a peripheral biomarker of treatment-resistant depression and the rapid antidepressant response: a comparison of ketamine and ECT. J Affect Disord. (2015) 186:306–11. doi: 10.1016/j.jad.2015.06.033, PMID: 26275358

[34] Bauer ME Papadopoulos A Poon L Perks P Lightman SL Checkley S. Altered glucocorticoid immunoregulation in treatment resistant depression. Psychoneuroendocrinology. (2003) 28:49–65. doi: 10.1016/s0306-4530(02)00009-4, PMID: 12445836

[35] Bauer M Papadopoulos A Poon L Perks P Lightman S Checkley S. Dexamethasone-induced effects on lymphocyte distribution and expression of adhesion molecules in treatment-resistant depression. Psychiatry Res. (2002) 113:1–15. doi: 10.1016/s0165-1781(02)00243-3 12467941

[36] Carpenter LL Bayat L Moreno F Kling MA Price LH Tyrka AR. Decreased cerebrospinal fluid concentrations of substance P in treatment-resistant depression and lack of alteration after acute adjunct vagus nerve stimulation therapy. Psychiatry Res. (2008) 157:123–9. doi: 10.1016/j.psychres.2007.04.016 17976740

[37] Cattaneo A Ferrari C Turner L Mariani N Enache D Hastings C. Whole-blood expression of inflammasome- and glucocorticoid-related mRNAs correctly separates treatment-resistant depressed patients from drug-free and responsive patients in the BIODEP study. Transl Psychiatry. (2020) 10:232. doi: 10.1038/s41398-020-00874-7, PMID: 32699209 PMC7376244

[38] Chamberlain SR Cavanagh J de Boer P Mondelli V Jones DNC Drevets WC. Treatment-resistant depression and peripheral C-reactive protein. Br J Psychiatry. (2019) 214:11–9. doi: 10.1192/bjp.2018.66, PMID: 29764522 PMC6124647

[39] Congio AC Norcia M Urbano MR Verri WA Vargas Nunes SO. Association of clinical features and biomarkers with treatment-resistant depression. Neurol Psychiatry Brain Res. (2020) 36:32–8. doi: 10.1016/j.npbr.2020.02.004

[40] de Menezes Galvão AC de Almeida RN Silva EADS Freire FAM Palhano-Fontes F Onias H. Cortisol modulation by Ayahuasca in patients with treatment resistant depression and healthy controls. Front Psych. (2018) 9:185. doi: 10.3389/fpsyt.2018.00185, PMID: 29867608 PMC5952178

[41] Galvao-Coelho NL de Menezes Galvao AC de Almeida RN Palhano-Fontes F Braga IC Soares BL. Changes in inflammatory biomarkers are related to the antidepressant effects of Ayahuasca. J Psychopharmacol. (2020) 34:1125–33. doi: 10.1177/0269881120936486, PMID: 32648790

[42] Gur S Taler M Bormant G Blattberg D Nitzan U Vaknin-Dembinsky A. Lack of association between unipolar or bipolar depression and serum aquaporin-4 autoantibodies. Brain Behav Immun. (2020) 88:930–4. doi: 10.1016/j.bbi.2020.05.001, PMID: 32380273

[43] Hoekstra R Van den Broek WW Fekkes D Bruijn JA Mulder PGH Pepplinkhuizen L. Effect of electroconvulsive therapy on biopterin and large neutral amino acids in severe, medication-resistant depression. Psychiatry Res. (2001) 103:115–23. doi: 10.1016/s0165-1781(01)00282-7, PMID: 11549400

[44] Huang MH Chen MH Tu PC Bai YM Su TP Yang BH. Elevated tumor necrosis factor-alpha receptor subtype 1 and the association with abnormal brain function in treatment-resistant depression. J Affect Disord. (2018) 235:250–6. doi: 10.1016/j.jad.2018.04.037 29660639

[45] Juruena MF Pariante CM Papadopoulos AS Poon L Lightman S Cleare AJ. The role of mineralocorticoid receptor function in treatment-resistant depression. J Psychopharmacol. (2013) 27:1169–79. doi: 10.1177/0269881113499205 23904409

[46] Lauden A Geishin A Merzon E Korobeinikov A Green I Golan-Cohen A. Higher rates of allergies, autoimmune diseases and low-grade inflammation markers in treatment-resistant major depression. Brain Behav Immun Health. (2021) 16:100313. doi: 10.1016/j.bbih.2021.100313, PMID: 34589804 PMC8474658

[47] Maes M de Meester I Verkerk R de Medts P Wauters A Vanhoof G. Lower serum dipeptidyl peptidase IV activity in treatment resistant major depression: relationships with immune-inflammatory markers. Psychoneuroendocrinology. (1997) 22:65–78. doi: 10.1016/s0306-4530(96)00040-6, PMID: 9149329

[48] Maes M Vandoolaeghe E Neels H Demedts P Wauters A Meltzer HY. Lower serum zinc in major depression is a sensitive marker of treatment resistance and of the immune/inflammatory response in that illness. Biol Psychiatry. (1997) 42:349–58. doi: 10.1016/S0006-3223(96)00365-4, PMID: 9276075

[49] Maes M Bosmans E De Jongh R Kenis G Vandoolaeghe E Neels H. Increased serum IL-6 and IL-1 receptor antagonist concentrations in major depression and treatment resistant depression. Cytokine. (1997) 9:853–8. doi: 10.1006/cyto.1997.0238, PMID: 9367546

[50] Maes M Mihaylova I Kubera M Uytterhoeven M Vrydags N Bosmans E. Lower plasma coenzyme Q10 in depression: a marker for treatment resistance and chronic fatigue in depression and a risk factor to cardiovascular disorder in that illness. Neuro Endocrinol Lett. (2009) 30:462–9. 20010493

[51] Markopoulou K Papadopoulos A Juruena MF Poon L Pariante CM Cleare AJ. The ratio of cortisol/DHEA in treatment resistant depression. Psychoneuroendocrinology. (2009) 34:19–26. doi: 10.1016/j.psyneuen.2008.08.004 18805642

[52] Nasca C Bigio B Lee FS Young SP Kautz MM Albright A. Acetyl-l-carnitine deficiency in patients with major depressive disorder. Proc Natl Acad Sci U S A. (2018) 115:8627–32. doi: 10.1073/pnas.1801609115 30061399 PMC6112703

[53] Pisoni A Strawbridge R Hodsoll J Powell TR Breen G Hatch S. Growth factor proteins and treatment-resistant depression: a place on the path to precision. Front Psych. (2018) 9:386. doi: 10.3389/fpsyt.2018.00386 PMC6115516 30190686

[54] Rengasamy M McClain L Gandhi P Segreti AM Brent D Peters D. Associations of plasma interleukin-6 with plasma and cerebrospinal fluid monoamine biosynthetic pathway metabolites in treatment-resistant depression. Neurol Psychiatry Brain Res. (2018) 30:39–46. doi: 10.1016/j.npbr.2018.05.001

[55] Sánchez-Carro Y de la Torre-Luque A Leal-Leturia I Salvat-Pujol N Massaneda C de Arriba-Arnau A. Importance of immunometabolic markers for the classification of patients with major depressive disorder using machine learning. Prog Neuro-Psychopharmacol Biol Psychiatry. (2023) 121:110674. doi: 10.1016/j.pnpbp.2022.110674, PMID: 36332700

[56] Sasaki T Hashimoto K Oda Y Ishima T Yakita M Kurata T. Increased serum levels of oxytocin in 'Treatment resistant depression in adolescents (TRDIA)' group. PLoS One. (2016) 11:e0160767. doi: 10.1371/journal.pone.0160767, PMID: 27536785 PMC4990411

[57] Schwieler L Samuelsson M Frye MA Bhat M Schuppe-Koistinen I Jungholm O. Electroconvulsive therapy suppresses the neurotoxic branch of the kynurenine pathway in treatment-resistant depressed patients. J Neuroinflammation. (2016) 13:51. doi: 10.1186/s12974-016-0517-7 26925576 PMC4772340

[58] Sowa-Kucma M Styczen K Siwek M Misztak P Nowak RJ Dudek D. Lipid peroxidation and immune biomarkers are associated with major depression and its phenotypes, including treatment-resistant depression and melancholia. Neurotox Res. (2018) 33:448–60. doi: 10.1007/s12640-017-9835-5 29103192 PMC5766730

[59] Strawbridge R Jamieson A Hodsoll J Ferrier IN McAllister-Williams RH Powell TR. The role of inflammatory proteins in anti-glucocorticoid therapy for treatment-resistant depression. J Clin Med. (2021) 10:784. doi: 10.3390/jcm10040784, PMID: 33669254 PMC7920038

[60] Strawbridge R Hodsoll J Powell TR Hotopf M Hatch SL Breen G. Inflammatory profiles of severe treatment-resistant depression. J Affect Disord. (2019) 246:42–51. doi: 10.1016/j.jad.2018.12.037, PMID: 30578945

[61] Szałach ŁP Cubała WJ Lisowska KA. Changes in T-cell subpopulations and cytokine levels in patients with treatment-resistant depression-a preliminary study. Int J Mol Sci. (2022) 24:479. doi: 10.3390/ijms24010479, PMID: 36613927 PMC9820349

[62] Uint L Bastos GM Thurow HS Borges JB Hirata TDC França JID. Increased levels of plasma IL-1b and BDNF can predict resistant depression patients. Rev Assoc Med Bras. (2019) 65:361–9. doi: 10.1590/1806-9282.65.3.361, PMID: 30994834

[63] Vandoolaeghe E Maes M Vandevyvere J Neels H. Hypothalamic-pituitary-thyroid-axis function in treatment resistant depression. J Affect Disord. (1997) 43:143–50. doi: 10.1016/s0165-0327(96)01426-7, PMID: 9165383

[64] Van Hunsel F Wauters A Vandoolaeghe E Neels H Demedts P Maes M. Lower total serum protein, albumin, and beta-and gamma-globulin in major and treatment-resistant depression: effects of antidepressant treatments. Psychiatry Res. (1996) 65:159–69. doi: 10.1016/s0165-1781(96)03010-7, PMID: 9029664

[65] Wu X Dai B Yan F Chen Y Xu Y Xia Q. Serum cortisol, Nesfatin-1, and IL-113: potential diagnostic biomarkers in elderly patients with treatment-resistant depression. Clin Interv Aging. (2022) 17:567–76. doi: 10.2147/CIA.S361459, PMID: 35480963 PMC9038158

[66] Zhou Y Zheng W Liu W Wang C Zhan Y Li H. Antidepressant effect of repeated ketamine administration on kynurenine pathway metabolites in patients with unipolar and bipolar depression. Brain Behav Immun. (2018) 74:205–12. doi: 10.1016/j.bbi.2018.09.007 30213652

[67] Zincir S Öztürk P Bilgen AE Izci F Yükselir C. Levels of serum immunomodulators and alterations with electroconvulsive therapy in treatment-resistant major depression. Neuropsychiatr Dis Treat. (2016) 12:1389–96. doi: 10.2147/NDT.S106652, PMID: 27366071 PMC4913984

[68] Kendler KS. Incremental advances in psychiatric molecular genetics and nosology. World Psychiatry. (2022) 21:415–6. doi: 10.1002/wps.20999, PMID: 36073696 PMC9453899

[69] Penninx BWJH. Psychiatric symptoms and cognitive impairment in "long COVID": the relevance of immunopsychiatry. World Psychiatry. (2021) 20:357–8. doi: 10.1002/wps.20913, PMID: 34505378 PMC8429338

[70] Keshavan MS. Characterizing transdiagnostic premorbid biotypes can help progress in selective prevention in psychiatry. World Psychiatry. (2021) 20:231–2. doi: 10.1002/wps.20857, PMID: 34002515 PMC8129835

[71] Wakefield JC. Klerman’s “credo” reconsidered: neo-Kraepelinianism, Spitzer’s views, and what we can learn from the past. World Psychiatry. (2022) 21:4–25. doi: 10.1002/wps.20942, PMID: 35015356 PMC8751581

[72] Arango C Dragioti E Solmi M Cortese S Domschke K Murray RM. Risk and protective factors for mental disorders beyond genetics: an evidence-based atlas. World Psychiatry. (2021) 20:417–36. doi: 10.1002/wps.20894, PMID: 34505386 PMC8429329

[73] Islam MR Sohan M Daria S Masud AA Ahmed MU Roy A. Evaluation of inflammatory cytokines in drug-naïve major depressive disorder: a systematic review and meta-analysis. Int J Immunopathol Pharmacol. (2023) 37:3946320231198828. doi: 10.1177/03946320231198828, PMID: 37625799 PMC10467201

[74] Lanquillon S Krieg JC Bening-Abu-Shach U Vedder H. Cytokine production and treatment response in major depressive disorder. Neuropsychopharmacology. (2000) 22:370–9. doi: 10.1016/S0893-133X(99)00134-7 10700656

[75] Uher R Tansey KE Dew T Maier W Mors O Hauser J. An inflammatory biomarker as a differential predictor of outcome of depression treatment with escitalopram and nortriptyline. Am J Psychiatry. (2014) 171:1278–86. doi: 10.1176/appi.ajp.2014.14010094 25017001

[76] Zhu CB Blakely RD Hewlett WA. The proinflammatory cytokines interleukin-1beta and tumor necrosis factor-alpha activate serotonin transporters. Neuropsychopharmacology. (2006) 31:2121–31. doi: 10.1038/sj.npp.1301029, PMID: 16452991

[77] Haroon E Daguanno AW Woolwine BJ Goldsmith DR Baer WM Wommack EC. Antidepressant treatment resistance is associated with increased inflammatory markers in patients with major depressive disorder. Psychoneuroendocrinology. (2018) 95:43–9. doi: 10.1016/j.psyneuen.2018.05.026, PMID: 29800779 PMC6427066

[78] Krueger RF Hobbs KA Conway CC Dick DM Dretsch MN Eaton NR. Validity and utility of hierarchical taxonomy of psychopathology (HiTOP): II. Externalizing superspectrum. World Psychiatry. (2021) 20:171–93. doi: 10.1002/wps.20844, PMID: 34002506 PMC8129870

[79] de Felice G Luciano M Boiano A Colangelo G Catapano P Della Rocca B. Can brain-derived neurotrophic factor be considered a biomarker for bipolar disorder? An analysis of the current evidence. Brain Sci. (2023) 13:1221. doi: 10.3390/brainsci13081221, PMID: 37626577 PMC10452328

[80] Lydiard RB. Worried sick: antidepressants, stress, and inflammation. J Clin Psychiatry. (2007) 68:1613–4. doi: 10.4088/jcp.v68n1021 17960979

[81] Felger JC Lotrich FE. Inflammatory cytokines in depression: neurobiological mechanisms and therapeutic implications. Neuroscience. (2013) 246:199–229. doi: 10.1016/j.neuroscience.2013.04.060, PMID: 23644052 PMC3741070

[82] Laugesen K Farkas DK Vestergaard M Jørgensen JOL Petersen I Sørensen HT. Glucocorticoid use and risk of suicide: a Danish population-based case-control study. World Psychiatry. (2021) 20:142–3. doi: 10.1002/wps.20831, PMID: 33432747 PMC7801823

[83] Feldman R. What is resilience: an affiliative neuroscience approach. World Psychiatry. (2020) 19:132–50. doi: 10.1002/wps.20729, PMID: 32394561 PMC7215067

[84] Lee DH Lee JY Hong DY Lee EC Park SW Lee YK. Pharmacological treatment for Neuroinflammation in stress-related disorder. Biomedicine. (2022) 10:2518. doi: 10.3390/biomedicines10102518, PMID: 36289780 PMC9599149

[85] Dinan TG Cryan JF. Gut microbiota: a missing link in psychiatry. World Psychiatry. (2020) 19:111–2. doi: 10.1002/wps.20726, PMID: 31922692 PMC6953563

[86] Zádor F Joca S Nagy-Grócz G Dvorácskó S Szűcs E Tömböly C. Pro-inflammatory cytokines: potential links between the endocannabinoid system and the kynurenine pathway in depression. Int J Mol Sci. (2021) 22:5903. doi: 10.3390/ijms22115903, PMID: 34072767 PMC8199129

[87] Cavaleri D Bartoli F Capogrosso CA Guzzi P Moretti F Riboldi I. Blood concentrations of neopterin and biopterin in subjects with depression: a systematic review and meta-analysis. Prog Neuro-Psychopharmacol Biol Psychiatry. (2023) 120:110633. doi: 10.1016/j.pnpbp.2022.110633 36089162

[88] Kalkman HO Feuerbach D. Antidepressant therapies inhibit inflammation and microglial M1-polarization. Pharmacol Ther. (2016) 163:82–93. doi: 10.1016/j.pharmthera.2016.04.001 27101921

[89] Veronese N Stubbs B Solmi M Ajnakina O Carvalho AF Maggi S. Acetyl-L-carnitine supplementation and the treatment of depressive symptoms: a systematic review and Meta-analysis. Psychosom Med. (2018) 80:154–9. doi: 10.1097/PSY.0000000000000537, PMID: 29076953

[90] Dyck MJ. Treatment-resistant depression: a critique of current approaches. Aust N Z J Psychiatry. (1994) 28:34–41. doi: 10.3109/00048679409075843, PMID: 8067966

[91] Conway CR George MS Sackeim HA. Toward an evidence-based, operational definition of treatment-resistant depression: when enough is enough. JAMA. Psychiatry. (2017) 74:9–10. doi: 10.1001/jamapsychiatry.2016.2586, PMID: 27784055

[92] Borsboom D Haslbeck JMB Robinaugh DJ. Systems-based approaches to mental disorders are the only game in town. World Psychiatry. (2022) 21:420–2. doi: 10.1002/wps.21004, PMID: 36073701 PMC9453900

